# Incidence of invasive fungal infection in acute lymphoblastic and acute myelogenous leukemia in the era of antimold prophylaxis

**DOI:** 10.1038/s41598-021-01716-2

**Published:** 2021-11-12

**Authors:** Sang-Min Oh, Ja Min Byun, Euijin Chang, Chang Kyung Kang, Dong-Yeop Shin, Youngil Koh, Junshik Hong, Taek Soo Kim, Pyoeng Gyun Choe, Wan Beom Park, Nam Joong Kim, Sung-Soo Yoon, Inho Kim, Myoung-don Oh

**Affiliations:** 1 Department of Internal Medicine, Seoul National University College of Medicine, Seoul, Republic of Korea; 2 Cancer Research Institute, Seoul National University College of Medicine, Seoul, Republic of Korea; 3 Department of Laboratory Medicine, Seoul National University College of Medicine, Seoul, Republic of Korea

**Keywords:** Fungal infection, Acute lymphocytic leukaemia, Acute myeloid leukaemia, Risk factors

## Abstract

The incidence of invasive fungal infection (IFI) in patients with acute myeloid leukemia (AML) has decreased with the introduction of antimold prophylaxis. Although acute lymphoblastic leukemia (ALL) has a lower risk of IFI than does AML, the incidences of IFI in both AML and ALL in the era of antimold prophylaxis should be re-evaluated. We analyzed adults with AML or ALL who had undergone induction, re-induction, or consolidation chemotherapy from January 2017 to December 2019 at Seoul National University Hospital. Their clinical characteristics during each chemotherapy episode were reviewed, and cases with proven or probable diagnoses were regarded as positive for IFI. Of 552 episodes (393 in AML and 159 in ALL), 40 (7.2%) were IFI events. Of the IFI episodes, 8.1% (12/148) and 5.9% (13/220) (*P* = 0.856) occurred in cases of ALL without antimold prophylaxis and AML with antimold prophylaxis, respectively. After adjusting for clinical factors, a lack of antimold prophylaxis (adjusted odds ratio [aOR], 3.52; 95% confidence interval [CI], 1.35–9.22; *P* = 0.010) and a longer duration of neutropenia (per one day, aOR, 1.02; 95% CI, 1.01–1.04; *P* = 0.001) were independently associated with IFI. In conclusion, the incidence of IFI in ALL without antimold prophylaxis was not lower than that in AML. A lack of antimold prophylaxis and prolonged neutropenia were independent risk factors for IFI. Clinicians should be on guard for detecting IFI in patients with ALL, especially those with risk factors.

## Introduction

Invasive fungal infections (IFIs) are opportunistic infections that cause substantial morbidity and mortality in patients with acute leukemia. Classically, the risk of IFI has been considered higher in acute myelogenous leukemia (AML) than in acute lymphoblastic leukemia (ALL), possibly due to leukocyte dysfunctions in the myeloid lineage and a longer duration of neutropenia following cytotoxic chemotherapy^1–5^.

There are no specific recommendations for antifungal prophylaxis in cytotoxic chemotherapy for ALL, partly due to the lower incidence of IFI^6^. By contrast, posaconazole prophylaxis is now routinely recommended in induction or re-induction chemotherapy for patients with AML^6^, because it significantly reduces the incidence of IFI in those patients^1^.

The incidences of IFI in patients with AML and ALL with or without antimold prohpylaxis needs to be re-evaluated to guide clinicians what level of suspicion for IFI is warranted in these respective scenarios. Specifically, the incidences of IFI in ALL without prophylaxis and AML with prophylaxis are of interest to assess whether ALL still has a lower risk of IFI compared to AML in the era of antimold prophylaxis.

Therefore, we elucidated the incidence of, and risk factors for IFI in patients with AML or ALL receiving cytotoxic chemotherapy after the introdction of posaconazole prophylaxis for AML.

## Results

### Clinical characteristics of patients with AML or ALL

A total of 552 episodes were identified for 149 AML (393 episodes) and 51 ALL (159 episodes) patients (Table 1). The patients with AML episodes were significantly older (median {interquartile range [IQR]}) (56 [49–63] years for AML patients *vs.* 45 [31–62] years for ALL patients, *P* < 0.001) and had higher CCI scores (3 [3–5] for AML patients *vs.* 3 [2–4] for ALL patients, *P* < 0.001) and a longer duration of neutropenia (18 [11–28] days for AML patients *vs.* 8 [3–15] days for ALL patients, *P* < 0.001). Induction or re-induction episodes were significantly more frequent in the AML patients than in the ALL patients (237/393 [60.3%] for AML patients *vs.* 73/159 [45.9%] for ALL patients, *P* = 0.002), and the rate of antimold prophylaxis was significantly higher for AML episodes (220/393 [56.0%] for AML episodes *vs.* 11/159 [6.9%] for ALL episodes, *P* < 0.001). Posaconazole was the most commonly used prophylactic agent, which was used for durations of (days, median [IQR]) of 25 (18–34) in AML and 8 (6–8) in ALL.

**Table 1:** Baseline characteristics of all episodes of acute leukemia.

Characteristics	AML episodes (n = 393)	ALL episodes (n = 159)	*P*
Age, median (IQR)	56 (49–63)	45 (31–62)	< 0.001
Male	205 (52.2)	73 (45.9)	0.183
CCI score, median (IQR)	3 (3–5)	3 (2–4)	< 0.001
Induction or re-induction chemotherapy (*vs.* consolidation)	237 (60.3)	73 (45.9)	0.002
Antimold prophylaxis	220 (56.0)	11 (6.9)	< 0.001
Posaconazole	217 (55.2)	5 (3.1)	
Others	3 (0.8)*	6 (3.8)^†^	
Duration of neutropenia, median, IQR	18 (11–28)	8 (3–15)	< 0.001
IFI episodes	28 (7.1)	12 (7.5)	0.862
Invasive aspergillosis	21 (5.3)^‡^	7 (4.4)	
Invasive candidiasis	8 (2.0)^‡^	5 (3.1)	
Others	1 (0.3)^§^	0 (0.0)	

Data are shown as number (%), not otherwise specified.

*Other prophylactic agents included 3 micafungin.

^†^Other prophylactic agents included 3 itraconazole and 3 micafungin.

^‡^Two episodes had IA and IC simultaneously.

^§^One cryptococcosis.

Comparing the clinical characteristics between episodes with and without antimold prophylaxis, there were significant differences in CCI score, rate of induction or re-induction chemotherapy, type of leukemia, and duration of neutropenia (Supplementary Table S1).

### Incidence of IFI

The incidence of IFI was not significantly different between AML and ALL episodes (28/393 [7.1%] for AML episodes *vs.* 12/159 [7.5%] for ALL episodes, *P* = 0.862). Of all episodes with IFI, invasive aspergillosis was the most frequent type (21/28 [75.0%] for AML episodes and 7/12 [58.3%] for ALL episodes), followed by invasive candidiasis. 27 of 28 invasive aspergillosis cases in the present study met the criteria for a probable diagnosis.

We examined the incidence of IFI in AML or ALL episodes with or without antimold prophylaxis (Table 2). The incidence of IFI in AML episodes with antimold prophylaxis was 5.9% (13/220) versus 8.7% (15/173) without. In ALL episodes, the incidence of IFI was 0% (0/11) with antimold prophylaxis and 8.1% (12/148) without. The difference in IFI incidence between ALL episodes without antimold prophylaxis and AML episodes with antimold prophylaxis was not significant (OR, 1.41; 95% CI, 0.62–3.17; *P* = 0.856). When the latest 2020 European Organization for Research and Treatment of Cancer and the Mycoses Study Group Education and Research Consortium (EORTC/MSGERC) definition was used (Supplementary Table S2), the incidences of IFI were 5.0% (11/220) in AML with prophylaxis and 7.5% (13/173) in ALL without prophylaxis.

**Table 2:** Incidence of invasive fungal infections in acute lymphoblastic or acute myelogenous leukemia with or without prophylactic antimold agents.

	IFI episodes	OR (95% CI) (*vs.* AML with prophylaxis)
AML without prophylaxis (n = 173)	15 (8.7)	1.51 (0.70–3.27)*
ALL without prophylaxis (n = 148)	12 (8.1)	1.41 (0.62–3.17)*
AML with prophylaxis (n = 220)	13 (5.9)	–
ALL with prophylaxis (n = 11)	0 (0.0)	NA

* *P* > 0.05.

We also evaluated the incidence of invasive mold infection in various scenarios (Supplementary Table S3). All of these were cases of invasive pulmonary aspergillosis. The incidence in AML episodes with antimold prophylaxis was 4.1% (9/220) versus 6.9% (12/173) without. The incidence of invasive mold infection in ALL episodes with antimold prophylaxis was 0% (0/11) versus 4.7% (7/148) without.

### Independent risk factors for IFI

Uni- and multivariable analyses were performed to compare episodes with and without IFI to elucidate independent risk factors for IFI (Table 3). In the univariable analysis, there were significant differences in the rate of induction or re-induction chemotherapy (29/40 [72.5%] for IFI episodes *vs.* 281/512 [54.9%] for non-IFI episodes, *P* = 0.031) and the number of days of neutropenia (median [IQR], 21 [12–32] for IFI episodes *vs.* 14.5 [8–25] for non-IFI episodes, *P* = 0.006). Multivariable analysis revealed that a lack of antimold prophylaxis (adjusted OR [95% CI], 3.52 [1.35–9.22]; *P* = 0.01) and a longer duration of neutropenia (adjusted OR [95% CI], per day, 1.02 [1.01–1.04]; *P* = 0.004) were independent risk factors for IFI. The results were similar when the 2020 EORTC/MSGERC definition was applied (Supplementary Table S4).

**Table 3:** Uni- and multi-variable analysis for risk factors of invasive fungal infection.

Characteristics	IFI episodes (n = 40)	Non-IFI episodes (n = 512)	Univariable analysis	Multivariable analysis	
			*P*	aOR (95% CI)	*P*
Age, median (IQR)	58 (41–65)	54 (43–62)	0.052	1.01 (0.96–1.06)	0.768
Male	15 (37.5)	263 (51.4)	0.091	0.69 (0.28–1.70)	0.422
CCI score, median (IQR)	3 (2–5)	3 (2–4)	0.136	1.05 (0.74–1.48)	0.805
AML (vs. ALL)	28 (70.0)	365 (71.3)	0.862	1.30 (0.32–5.19)	0.714
Induction or re-induction chemotherapy (*vs.* consolidation)	29 (72.5)	281 (54.9)	0.031	1.79 (0.71–4.52)	0.220
No antimold prophylaxis	27 (67.5)	294 (57.4)	0.213	3.52 (1.35–9.22)	0.010
Duration of neutropenia, day, median (IQR)	21 (12–32)	15 (8–25)	0.006	1.02 (1.01–1.04)	0.001

Data are shown as number (%), not otherwise specified.

### Sensitivity analyses

Seventy-two episodes involving the therapeutic administration of antimold agents during the episodes were excluded for the first sensitivity analysis. The incidence of IFI in AML episodes with antimold prophylaxis was 7.4% (13/176) versus 9.0% (12/134) in ALL episodes without antimold prophylaxis (Supplementary Table S5; OR [95% CI], 1.23 [0.54–2.80]; *P* = 0.616). Independent risk factors for IFI were a lack of antimold prophylaxis (adjusted OR [95% CI], 3.77 [1.32–10.79]; *P* = 0.013) and prolonged neutropenia (adjusted OR [95% CI], per one day, 1.03 [1.01–1.04]; *P* = 0.001) (Supplementary Table S6).

The 200 episodes (all were induction or re-induction) included in the second sensitivity analysis, included 11 IFI episodes (Supplementary Table S7). In patients with AML, the incidence of IFI was 4.9% (7/144) with prophylaxis versus 20.0% (1/5) without. In patients with ALL, the incidence was 0% (0/6) and 6.7% (3/45), respectively. Only a longer duration of neutropenia was an independent risk factor for IFI in this subgroup (adjusted OR [95% CI], 1.03 [1.01–1.06]; *P* = 0.015) (Supplementary Table S8).

## Discussion

Although AML has been traditionally believed to be associated with a greater risk of IFI than ALL^2–5^, the recent introduction of antimold prophylaxis in induction and re-induction chemotherapy has considerably reduced the incidence of IFI in AML^1,6,7^. Since then, it was unknown whether ALL is still associated with a lower risk of IFI than AML in the era of antimold prophylaxis. The study was performed to address this, and we found that the incidence of IFI in ALL was not lower than that in AML.

In addition to previous observations that AML is associated with a higher incidence of IFI than is ALL^8,9^, there are some theoretical reasons for a higher risk of IFI in AML than in ALL. First, myeloid cell dysfunction poses a greater risk of IFI than does lymphoid cell dysfunction. Second, the duration of neutropenia after cytotoxic chemotherapy is generally longer in AML than in ALL^2,5,9^. In an effort to reduce IFI in AML, a multicenter randomized controlled trial of patients with AML or myelodysplastic syndrome was performed, which showed that posaconazole prophylaxis significantly reduced the incidence of IFI compared with fluconazole or itraconazole prophylaxis^1^. This led to the current recommendation for posaconazole prophylaxis in patients with AML undergoing induction or re-induction chemotherapy^6,10^.

No routine antimold prophylaxis is recommended for patients with ALL receiving cytotoxic chemotherapy, owing to the lower IFI incidence and possible neurotoxic interactions between azoles and vinca alkaloids^6,11^. Therefore, studies of the incidences of, and risk factors for IFI in AML or ALL that reflect the current situation are warranted to guide clinicians. Because the diagnosis of IFI is often complicated, estimating the pre-test probability of IFI in patients with acute leukemia is very important for interpreting the results of clinical and mycological tests^12–14^. We showed that the incidence of IFI in ALL without antimold prophylaxis was not lower than that in AML with antimold prophylaxis, indicating that the notion of a lower risk of IFI in ALL than in AML needs to be modified. Clinicians should remain highly suspicious of IFI in ALL patients who are not receiving antimold prophylaxis, especially those with prolonged neutropenia. In addition, antimold prophylaxis could be considered in patients with ALL who are expected to have a longer duration of neutropenia than usual occasion.

Studies suggest that an advanced age, prolonged neutropenia, and the relapse of hematological malignancies are risk factors for IFI in acute leukemia^2,5^. However, most studies were performed on AML cases, and few have examined the risk factors for IFI in patients with ALL. In a single-center, retrospective study, Nicolato et al*.* showed that a longer duration of neutropenia, the relapse of underlying diseases, a longer duration of hospitalization before the first fever, and allogeneic HSCT were risk factors for IFI in ALL^9^, which is in accordance with our results. Notably, the type of acute leukemia itself (*i.e.*, AML and ALL) was not independently associated with the occurrence of IFI in this study.

To increase the reliability of our results, sensitivity analyses were performed under two conditions. In the first, we excluded those who received a therapeutic antimold agent during an episode. In the second, we included only the first episodes of every patient in the study period, although we used a GEE to explore independent risk factors for IFI to lessen the effects of repeated inclusion of patients^15^. The main results, namely, that the incidence of IFI in ALL without prophylaxis was comparable to that in AML with prophylaxis, and a lack of antimold prophylaxis and a longer duration of neutropenia were independent risk factors for IFI, remained unchanged in these analyses (Supplementary Table S5–S8). In addition, the incidence of invasive mold infection did not differ significantly between these two groups (Supplementary Table S3).

There are some limitations to our study. First, it included a study population with a small sample size enrolled from a single university-affiliated hospital. Particularly, the analyses of the difference in IFI incidence according to the clinical scenario might have been under-powered. Second, because the diagnosis of IFI is often challenging and the majority of IFI cases in this study were only probable diagnoses, the retrospective nature of our study also needs to be considered. Lastly, we could not suggest a specific antimold prophylaxis regimen or schedule in ALL in this study. Further studies are warranted to validate our results and to examine clinical impact of antimold prophylaxis in selected patients with ALL. Therefore, a larger prospective study is warranted to validate our results.

In conclusion, the incidence of IFI in ALL episodes without antimold prophylaxis was not lower than that in AML episodes with antimold prophylaxis, and a lack of antimold prophylaxis and a longer duration of neutropenia were independent risk factors for IFI. Therefore, in the current era of antimold prophylaxis, clinicians should also be on guard for the occurrence of IFI in patients with ALL, especially those with characteristics that put them at higher risk.

## Materials and methods

### Study design and data collection

We retrospectively reviewed all adults (≥ 18 years) with AML or ALL who received induction, re-induction, or consolidation cytotoxic chemotherapy from January 2017 to December 2019 at Seoul National University Hospital. Cases of hematopoietic stem-cell transplantation (HSCT) were excluded, because antimold prophylaxis is routinely given regardless of the type of acute leukemia^6^. Episodes of palliative chemotherapy, such as that using decitabine or azacitidine, and research-based chemotherapy were also excluded.

Clinical data were reviewed using electronic medical records including those on age, gender, the type of acute leukemia, purpose of cytotoxic chemotherapy, specific agents administered, severity of underlying diseases measured by based on the Charlson’s comorbidity-weighted index (CCI) score^16^, administration of antimold agent, duration of neutropenia, and occurrence and type of IFI.

The incidence of IFI was examined in AML or ALL episodes with or without antimold prophylaxis. The clinical characteristics of the IFI episodes were compared to reveal independent risk factors for IFI.

This study was approved by the Institutional Review Board at Seoul National University Hospital (IRB no. 2008–167-1151), and they waived obtaining the informed consent because of the retrospective nature of the study. All study procedures were performed in accordance with the Declaration of Helsinki.

### Definition

Only cases with proven and probable diagnoses following the 2008 Revised EORTC/MSG guideline were regarded as positive for IFI in this study^17^. Although we used the 2008 version in main analysis to account for the study period preceding the guideline revision, and to retain a higher sensitivity for the clinical diagnosis of IFI^12^, we performed the same analyses and presented the results using 2020 version.

An episode was defined as the period between the date of starting chemotherapy and the date of discharge. If two or more rounds of chemotherapy were performed during one hospitalization, the period was divided according to the day the subsequent chemotherapy began^19^. Antimold prophylaxis included routine posaconazole prophylaxis for induction or re-induction chemotherapy in AML or antimold agents for secondary prophylaxis due to a history of invasive mold infection.

The duration of neutropenia was defined as the time between the first date of an absolute neutrophil count (ANC) lower than 500/mm^3^ and the third day with an ANC higher than 500/mm^3^, as previously described^9,20,21^.

### Sensitivity analysis

Two sensitivity analyses were performed to account for potential confounders. First, the incidences of, and risk factors for IFI were re-examined after excluding all episodes with therapeutic administration of an antimold agent (e.g., liposomal amphotericin B, caspofungin, etc.) for prolonged neutropenic fever, because this could obscure the effect of a prophylactic antimold agent. Second, we analyzed the subgroup that included only the first episodes for every patient to exclude the effects from repeated inclusion of the patients.

### Statistical analysis

The chi-square or Fisher’s exact test was used to compare categorical variables. Based the normality of continuous variables assessed using the Shapiro–Wilk test, the Mann–Whitney *U* test or Students’ *t-* test was used. To calculate odds ratio (OR) and associated 95% confidence interval (CI) for IFI in different clinical scenarios (AML or ALL with or without antimold prophylaxis) logistic regression was used. For the multivariable analysis, variables were analyzed using backward stepwise logistic regression. Variables were removed using a *P* value of 0.05 as the cut-off value. Where applicable, generalized estimating equations (GEEs) were used to account for the inclusion of multiple episodes from a patient^15^. A *P* value < 0.05 was considered to indicate statistical significance in all analyses. All statistical analysis were performed using IBM SPSS Statistics, ver. 27.0 (IBM, Armonk, NY, USA).

## Supplementary Information

Supplementary Information.
