# Supplementary material for: Incidence of invasive fungal infection in acute lymphoblastic and acute myelogenous leukemia in the era of antimold prophylaxis
Source: Sci Rep. 2021 Nov 12;11:22160. doi: 10.1038/s41598-021-01716-2 (PMC8590008; doi:10.1038/s41598-021-01716-2)
Supplement: Supplementary file 1 — Supplementary Information. [file 41598_2021_1716_MOESM1_ESM.docx]

| Characteristics | Antimold prophylaxis (n=231) | No antimold prophylaxis (n=321) | *P* |
| --- | --- | --- | --- |
| Age, median (IQR) | 56 (48-62) | 54 (40-63) | 0.106 |
| Male | 126 (54.5) | 152 (47.4) | 0.095 |
| CCI score, median (IQR) | 3 (3-4) | 3 (2-4) | 0.008 |
| Induction or re-induction chemotherapy (vs. consolidation) | 225 (97.4) | 85 (26.5) | <0.001 |
| AML (vs. ALL) | 220 (95.2) | 173 (53.9) | <0.001 |
| Duration of neutropenia, median (IQR) | 26 (19-33) | 10 (6-15) | <0.001 |
| IFI episodes | 13 (5.6) | 27 (8.4) | 0.213 |
| Invasive aspergillosis | 9 (3.9) | 19 (5.9)^*^ |  |
| Invasive candidiasis | 3 (1.3) | 10 (3.1)^*^ |  |
| Others | 1 (0.4) | 0 (0.0) |  |

Data are shown as number (%), not otherwise specified.

^*^Two episodes had IA and IC simultaneously.

**Supplementary Table S1. Baseline characteristics of all episodes according to the administration of antimold prophylaxis.**

|  | IFI episodes  (n=36) | OR (95% CI) (*vs.* AML with prophylaxis) |
| --- | --- | --- |
| AML without prophylaxis (n=173) | 13 (7.5) | 1.54 (0.67-3.54)^*^ |
| ALL without prophylaxis (n=148) | 12 (8.1) | 1.68 (0.72-3.91)^*^ |
| AML with prophylaxis (n=220) | 11 (5.0) | - |
| ALL with prophylaxis (n=11) | 0 (0.0) | NA |

^*^ *P* > 0.05

**Supplementary Table S2. Incidence of invasive fungal infections after applying definition of 2020 revised EORTC/MSGERC guideline.**

|  | IFI episodes | OR (95% CI) (*vs.* AML with prophylaxis) |
| --- | --- | --- |
| AML without prophylaxis (n=173) | 12 (6.9) | 1.75 (0.72-4.25)^*^ |
| ALL without prophylaxis (n=148) | 7 (4.7) | 1.16 (0.42-3.20)^*^ |
| AML with prophylaxis (n=220) | 9 (4.1) | - |
| ALL with prophylaxis (n=11) | 0 (0.0) | NA |

^*^ *P* > 0.05

**Supplementary Table S3. Incidence of invasive pulmonary aspergillosis in acute lymphoblastic or acute myelogenous leukemia with or without prophylactic antimold agents.**

| Characteristics | IFI episodes  (n=36) | Non-IFI episodes  (n=516) | Univariable analysis | Multivariable analysis | |
| --- | --- | --- | --- | --- | --- |
|  |  |  | *P* | aOR (95% CI) | *P* |
| Age, median (IQR) | 58 (36-65) | 54 (43-62) | 0.166 | 1.00 (0.95-1.05) | 0.945 |
| Male | 12 (33.3) | 266 (51.6) | 0.035 | 0.61 (0.24-1.55) | 0.297 |
| CCI score, median (IQR) | 3 (2-5) | 3 (2-4) | 0.332 | 1.05 (0.71-1.55) | 0.812 |
| AML (vs. ALL) | 24 (66.7) | 369 (71.5) | 0.535 | 1.13 (0.27-4.71) | 0.863 |
| Induction or re-induction chemotherapy (*vs.* consolidation) | 25 (69.4) | 285 (55.2) | 0.097 | 1.61 (0.63-4.14) | 0.322 |
| No antimold prophylaxis | 25 (69.4) | 296 (57.4) | 0.155 | 3.71 (1.33-10.36) | 0.012 |
| Duration of neutropenia, day, median (IQR) | 21 (11-37) | 15 (8-25) | 0.021 | 1.03 (1.01-1.04) | <0.001 |

Data are shown as number (%), not otherwise specified.

**Supplementary Table S4. Uni- and multi-variable analysis for risk factors of invasive fungal infection after applying definition of 2020 revised EORTC/MSGERC guideline.**

|  | IFI episodes  (n=40) | OR (95% CI) (*vs.* AML with prophylaxis) |
| --- | --- | --- |
| AML without prophylaxis (n=161) | 15 (9.3) | 1.29 (0.59-2.80)^*^ |
| ALL without prophylaxis (n=134) | 12 (9.0) | 1.23 (0.54-2.80)^*^ |
| AML with prophylaxis (n=176) | 13 (7.4) | - |
| ALL with prophylaxis (n=9) | 0 (0.0) | NA |

^*^ *P* > 0.05

**Supplementary Table S5. Incidence of invasive fungal infections after excluding those who received therapeutic antimold agent during an episode.**

| Characteristics | IFI episodes  (n=40) | Non-IFI episodes  (n=440) | Univariable analysis | Multivariable analysis | |
| --- | --- | --- | --- | --- | --- |
|  |  |  | *P* | aOR (95% CI) | *P* |
| Age, median (IQR) | 58 (41-65) | 54 (43-62) | 0.058 | 1.00 (0.95-1.05) | 0.956 |
| Male | 15 (37.5) | 218 (49.5) | 0.144 | 0.77 (0.31-1.88) | 0.559 |
| CCI score, median (IQR) | 3 (2-5) | 3 (2-4) | 0.126 | 1.09 (0.77-1.55) | 0.632 |
| AML (vs. ALL) | 28 (70.0) | 309 (70.2) | 0.976 | 1.50 (0.36-6.25) | 0.577 |
| Induction or re-induction chemotherapy (vs. consolidation) | 29 (72.5) | 218 (49.5) | 0.005 | 1.97 (0.69-5.63) | 0.206 |
| No antimold prophylaxis | 27 (67.5) | 268 (60.9) | 0.412 | 3.77 (1.32-10.79) | 0.013 |
| Duration of neutropenia, day, median (IQR) | 12 (12-32) | 13 (7-23) | 0.001 | 1.03 (1.01-1.04) | 0.001 |

Data are shown as number (%), not otherwise specified.

**Supplementary Table S6. Uni- and multivariable analysis for risk factors of invasive fungal infection after excluding those who received therapeutic antimold agent during an episode.**

|  | IFI episodes  (n=11) | OR (95% CI) (*vs.* AML with prophylaxis) |
| --- | --- | --- |
| AML without prophylaxis (n=5) | 1 (20.0) | 4.89 (0.48-49.75)^*^ |
| ALL without prophylaxis (n=45) | 3 (6.7) | 1.40 (0.35-5.65)^*^ |
| AML with prophylaxis (n=144) | 7 (4.9) | - |
| ALL with prophylaxis (n=6) | 0 (0.0) | NA |

^*^ *P* > 0.05

**Supplementary Table S7. Incidence of invasive fungal infections in the first episodes of each patient.**

| Characteristics | IFI episodes  (n=11) | Non-IFI episodes  (n=189) | Univariable analysis | Multivariable analysis | |
| --- | --- | --- | --- | --- | --- |
|  |  |  | *P* | aOR (95% CI) | *P* |
| Age, median (IQR) | 57 (33-65) | 55 (43-62) | 0.414 | - | 0.859 |
| Male | 4 (36.4) | 98 (51.9) | 0.318 | - | 0.509 |
| CCI score, median (IQR) | 3 (2-5) | 3 (2-4) | 0.496 | - | 0.933 |
| AML (vs. ALL) | 8 (72.7) | 141 (74.6) | 1.000 | - | 0.360 |
| No antimold prophylaxis | 4 (36.4) | 46 (24.3) | 0.472 | - | 0.123 |
| Duration of neutropenia, day, median (IQR) | 30 (26-43) | 24 (15-31) | 0.004 | 1.03 (1.01-1.06) | 0.015 |

Data are shown as number (%), not otherwise specified.

**Supplementary Table S8. Uni- and multivariable analyses for risk factors of invasive fungal infection in the first episodes of each patient**
